# Deep Learning‐Based Inclusion Boundary Identification Using Wave Propagation in Optical Coherence Elastography

**DOI:** 10.1002/jbio.70345

**Published:** 2026-08-23

**Authors:** Guangyu Zhang, Jinpeng Liao, Zhengshuyi Feng, Alison M. Layton, Chunhui Li, Zhihong Huang

**Affiliations:** ^1^ Healthcare Engineering, School of Physics and Engineering Technology, University of York York UK; ^2^ Biomedical Engineering, School of Science and Engineering, University of Dundee Dundee UK; ^3^ Hull York Medical School, University of York York UK

## Abstract

Elastography is widely used to estimate tissue mechanical properties based on wave propagation characteristics. However, a major limitation is that elastography maps often fail to accurately delineate internal boundaries within heterogeneous tissues. To address this issue, we propose a novel deep learning–based framework, termed Boundary Detection Network (BDNet). Depth‐resolved phase images capturing wave propagation are acquired using Optical Coherence Elastography (OCE). The proposed method is first evaluated on agar phantoms. Experimental results show that all evaluated models achieve comparable performance on single‐boundary samples, with a mean absolute error (MAE) of 2 pixels. For dual‐boundary phantoms, BDNet achieves an overall MAE of 4.33 pixels. Furthermore, on a human acne dataset, the proposed method provides the best performance, with an MAE of 19.11 pixels. The proposed BDNet demonstrates high accuracy and computational efficiency, providing a promising tool for assisting abnormal tissue morphology identification in clinical elastography applications.

## Graphical abstract

This study presents a deep learning framework for direct interface localization in OCE. The proposed BDNet enables accurate and efficient boundary detection, facilitating tissue morphology assessment in clinical elastography.

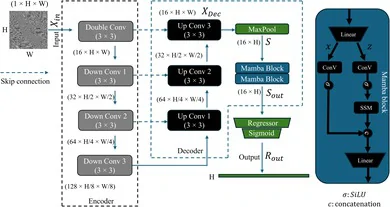

## 1 Introduction

Optical Coherence Elastography (OCE), as an extension of Optical Coherence Tomography (OCT), enables the estimation of tissue mechanical properties and has been widely applied in dermatology [1, 2, 3, 4], pathology [5, 6, 7], and ophthalmology [8, 9, 10]. In vivo, OCE has been used to assess tissue biomechanics in skin [11, 12], including pathological conditions such as acne [13], where tissue stiffness serves as a key biomarker for disease progression and treatment response [14].

In OCE, an external force is applied to induce mechanical wave propagation along the tissue surface and the temporal phase variations are subsequently analyzed to estimate wave velocity. Tissue stiffness is typically inferred from wave propagation characteristics; specifically, wave velocity is positively correlated with tissue stiffness. Conventional velocity estimation methods, such as time‐of‐flight approaches in the spatiotemporal domain [15] and phase velocity estimation in the frequency–wavenumber domain [16], are widely used to estimate wave velocity. In addition, deep learning–based methods have been introduced to improve velocity estimation. For instance, Zhang and Liao et al. proposed VPNet to estimate depth‐resolved velocity distributions using phase images as input [17], while DenseNet‐based models have also been employed for velocity estimation in agar phantoms and human skin [18]. Transformer‐based approaches have further been explored to capture global dependencies [19], and PVNet has been proposed to estimate phase velocity in the frequency domain for layered samples [20]. Transformer‐based models further enhance the modelling of long‐range dependencies through self‐attention mechanisms, enabling improved global context understanding [21]. Although effective in capturing global context, Transformer‐based methods often suffer from high computational cost. Besides, convolutional neural networks (e.g., UNet [22] and DenseNet [23]) have been widely used for regression tasks due to their ability to extract hierarchical local features. CNN‐based methods are inherently limited in modelling long‐range dependencies. Recently, state space models (SSMs) such as Mamba and its vision variant (MambaVision) have been proposed to efficiently capture long‐range dependencies with reduced computational complexity, offering a promising alternative to traditional architectures for handling high‐dimensional sequential data [24], but their application in OCE remains largely unexplored.

However, these methods still suffer from several limitations. First, traditional boundary detection in elastography relies on velocity or stiffness maps, which often exhibit gradual changes without clear discontinuities at inclusion edges and boundary offset [25, 26]. This gradual variation introduces subjectivity in boundary identification, as operators must interpret the maps manually. Moreover, velocity maps are sensitive to estimation parameters, such as window size, smoothing, or interpolation to fill missing points, which can degrade accuracy, particularly for low‐resolution maps [27, 28, 29]. Third, the shape of tissue inclusions is typically irregular and varies across both depth and lateral dimensions. These limitations collectively hinder reliable and precise identification of tissue interfaces in OCE.

To address these challenges, we propose a novel framework for direct boundary detection from wave propagation data, rather than relying on intermediate velocity estimation. Specifically, phase data acquired from OCE (with lateral, depth, and time dimensions) are used as input to the proposed Boundary Detection Network (BDNet), which combines UNet for spatial feature extraction with Mamba for efficient long‐range dependency modelling. In addition, a Margin‐Aware Soft Asymmetric (MASA) loss function is introduced to improve boundary localization performance. Experimental results on single‐ and dual‐boundary agar phantoms, as well as human acne data, demonstrate that the proposed method achieves higher accuracy compared with existing baseline approaches. To the best of our knowledge, this is among the first studies to directly detect tissue boundaries from wave propagation patterns using a deep learning framework in OCE while potentially providing a fast and accurate solution for identifying abnormal tissue structures in clinical applications.

## 2 Methods

This section presents the architecture of the proposed BDNet, MASA loss function, along with the training strategy and evaluation metrics used for quantitative comparison with baseline methods.

### 2.1 BDNet Construction

**FIGURE 1:**
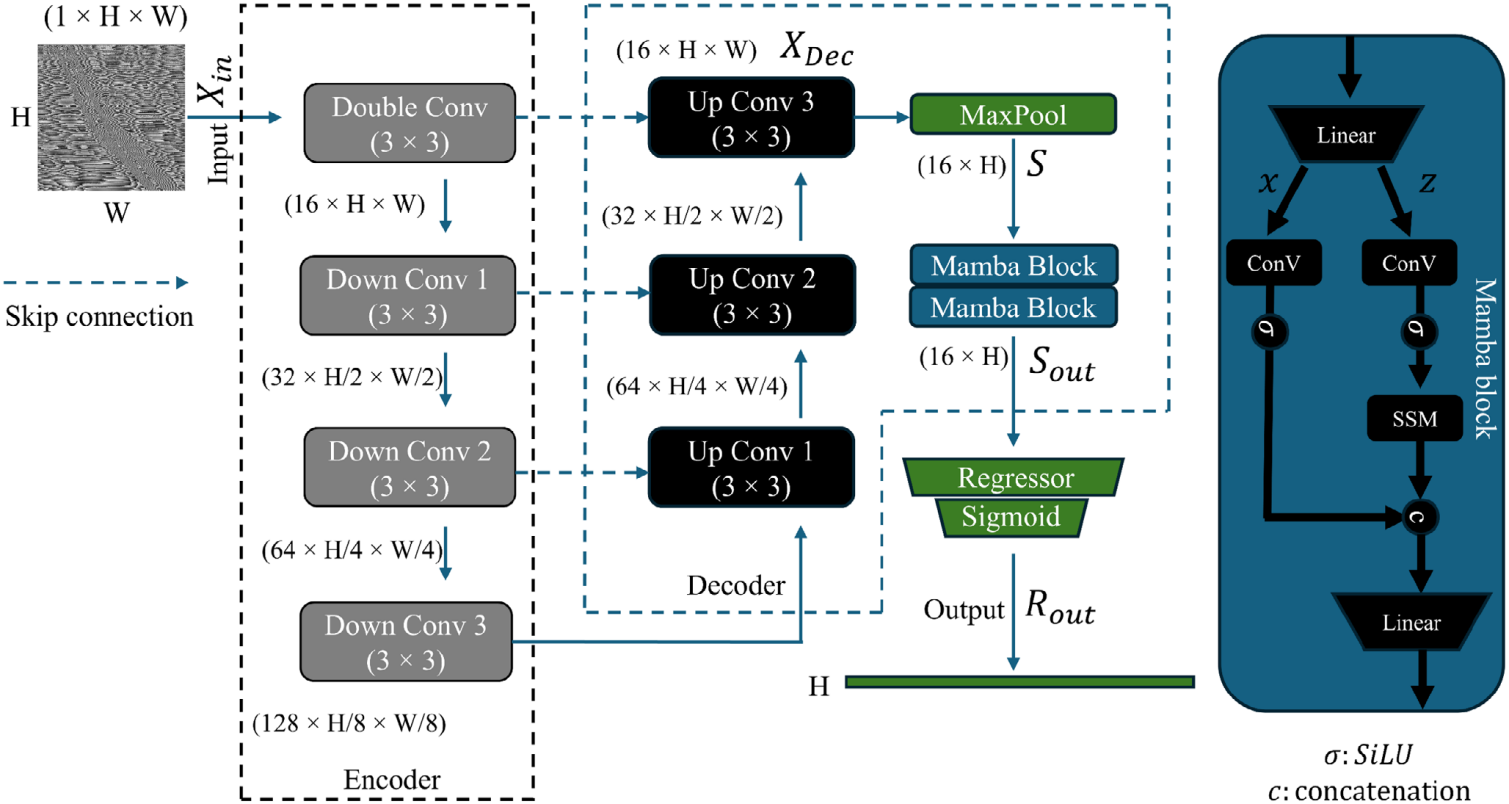
Overview of the proposed BDNet architecture, consisting of a UNet encoder–decoder structure and Mamba‐based sequence modelling modules. Skip connections (dashed blue lines) link encoder and decoder features via upsampling blocks. The detailed structure of the Mamba block is illustrated on the right.

The proposed model, BDNet, is based on a hybrid architecture that integrates a UNet backbone with a Mamba‐based SSM. It is specifically designed for continuous boundary localization from spatiotemporal OCE data, which leverages wave propagation dynamics encoded in the temporal dimension to infer boundary locations. To capture both local waveform variations and global boundary continuity, the model combines a convolutional encoder–decoder structure with a sequence modelling module. Given an input phase image $X_{\text{in}}\in R^{B\times 1\times H\times W}$, where H and W denote the lateral and temporal dimensions, and B is the batch size, the proposed network consists of four sequential stages: (1) Local Feature Extraction via UNet backbone, (2) Spatial‐to‐Sequence Dimension Reduction, (3) Global Dependency Modelling via 1D Mamba blocks, and (4) Coordinate Regression are shown in Figure 1.

#### 2.1.1 UNet Backbone

To extract multi‐scale spatial features, a lightweight UNet architecture is adopted as the backbone [22]. The encoder consists of an initial convolutional block followed by three downsampling stages. The decoder consists of three upsampling stages, each implemented using a 2 × 2 transposed convolution with Stride 2. Skip connections are introduced to concatenate encoder features with corresponding decoder features, preserving high‐resolution spatial information. After concatenation, a Double Conv block is applied to fuse features and reduce channel dimensions. The decoder outputs a feature map $X_{\mathrm{Dec}}\in R^{B\times C\times H\times W}$.

#### 2.1.2 Spatial‐to‐Sequence Dimension Reduction

A key challenge is transforming spatiotemporal wave propagation data into a compact representation for boundary localization. Given that boundary positions correspond to spatial discontinuities in wave propagation patterns, the 2D lateral–temporal representation is converted into a 1D sequence along the lateral dimension. Specifically, an Adaptive Max Pooling operation is applied along the temporal dimension (W) to aggregate the most salient wave responses at each spatial location. For the decoder output feature map $X_{\mathrm{Dec}}$, this operation extracts dominant propagation features along each lateral position, resulting in a sequence $S\in R^{B\times C\times H}$. This transformation preserves boundary‐related wave variations while significantly reducing computational complexity for subsequent sequence modelling.

#### 2.1.3 Mamba Block

The Mamba block adapts the SSM to enable efficient sequence modeling with linear computational complexity [30]. Given the 1D input tokens $X_{\text{in}}\in R^{B\times H\times C}$, it expands and splits equally the channels into a primary state branch (x) and a residual gating branch (z).

To capture local waveform patterns prior to global modelling, both branches are processed using depthwise 1D convolutions with a kernel size of 4, followed by a SiLU activation. The primary branch is then passed through SSM to model long‐range dependencies along the lateral dimension, which is critical for maintaining boundary continuity. The output of the SSM is fused with the gating branch via channel‐wise concatenation, followed by a linear projection:

(1) $$\begin{array}{c} S_{\text{out}}=\text{Linear}(\text{Concat}(\mathrm{SSM}\left(\sigma_{\text{SiLU}}\left(\text{Conv}1D_{k=4}\left(x\right)\right)\right),\\ \sigma_{\text{SiLU}}\left(\text{Conv}1D_{k=4}\left(z\right)\right))) \end{array}$$

#### 2.1.4 Coordinate Regression

The context‐enriched sequence is further processed by a 1D convolutional regressor to predict boundary locations. The regression head consists of a 1D convolution with kernel size of 3 followed by a GELU activation to refine local continuity, and a 1D convolution with kernel size of 1 to map the features to a single‐channel output. A Sigmoid function is applied to normalize the output:

(2) $$R_{\text{out}}=\text{Sigmoid}\left(\text{Conv}1D{\left(x\right)}_{k=1}\left(\sigma_{\text{GELU}}\left(\text{Conv}1D{\left(x\right)}_{k=3}\left(S_{\text{out}}\right)\right)\right)\right)$$

The output represents the probability distribution of boundary positions along the lateral dimension, corresponding to the locations of wave propagation discontinuities. The configurations of the BDNet variants (BDNet_S and BDNet_L) and baseline models are summarized in Tables S1–S5.

### 2.2 Margin Aware Soft Asymmetric (MASA) Loss function

To address the issues of missed boundaries and over‐penalization caused by background noise in boundary detection tasks, we propose a MASA loss function. This loss is designed to enhance sensitivity to boundary regions during training. Given the ground truth $y_{i}\in \left[0,1\right]$ and prediction $p_{i}\in \left[0,1\right]$, i is the index of pixel, the total loss L is defined as the sum of foreground loss $\left(L_{\mathrm{fg}}\right)$ and background loss $\left(L_{\mathrm{bg}}\right)$, the foreground corresponds to Gaussian labelled boundary regions, while the background includes all other areas.

(3) $$L=L_{\mathrm{fg}}+L_{\mathrm{bg}}$$

#### 2.2.1 Foreground Asymmetric Penalty

We introduce an asymmetric weighting mechanism in the foreground loss, including both missed boundaries and over‐predicted responses:

(4) $$L_{\mathrm{fg}}=\frac{1}{N_{\mathrm{fg}}}\sum_{i}y_{i}{\left(p_{i}-y_{i}\right)}^{2}\left[\lambda_{\text{miss}}\cdot I\left(p_{i}<y_{i}\right)+\lambda_{\text{false}}\cdot I\left(p_{i}\geq y_{i}\right)\right]$$

where $\lambda_{\text{miss}}$ is penalty for missed signal, $\lambda_{\text{false}}$ is penalty for false signal, I is indicator function (1 if the condition is satisfied, otherwise 0). In this study, we set $\lambda_{\text{miss}}=1$ and $\lambda_{\text{false}}=3$, prioritizing the preservation of weak boundary signals in multi‐boundary conditions. The normalization term is defined as $N_{\mathrm{fg}}=\max\left(\sum y_{i},1\right)$, which prevents trivial solutions and stabilizes training, fg indicates foreground.

#### 2.2.2 Background Suppression with Margin and Safe Zone

To suppress spurious responses in the background while avoiding excessive penalties near true boundaries, we introduce a double‐threshold mechanism for the background loss:

(5) $$L_{\mathrm{bg}}=\frac{1}{N_{\mathrm{bg}}}\sum_{i}\left(1-y_{i}\right){\left(p_{i}-y_{i}\right)}^{2}\cdot I\left(p_{i}>\tau_{\text{noise}}\right)\cdot I\left(y_{i}<\tau_{\text{safe}}\right)$$

where $\tau_{\text{noise}}$ is the lower threshold for background noise (set to 0.1) and $\tau_{\text{safe}}$ defines a safe margin near boundaries (set to 0.05). The normalization term, $N_{\mathrm{bg}}=\max\left(\sum \left(1-y_{i}\right),1\right)$, which helps maintain numerical stability during optimization, bg indicates background.

### 2.3 Pipeline of Model Training

**FIGURE 2:**
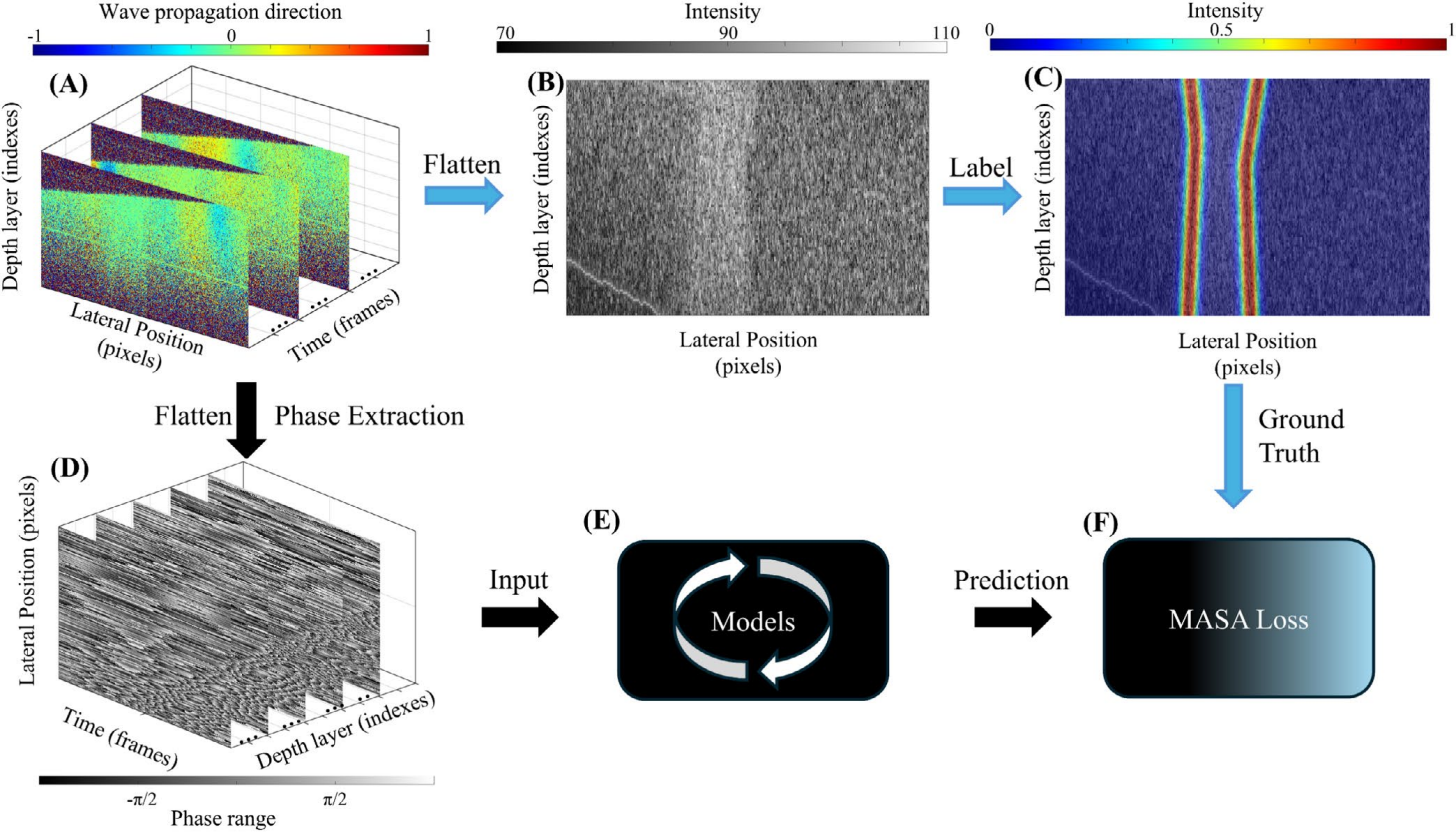
Training pipeline of the proposed method. (A) Acquired 3D OCE dataset (B) Structural B‐scan images after surface flattening. (C) Manually annotated boundary heatmaps overlaid on the corresponding B‐scan images. (D) Flattened phase dataset used as model input. (E) Training process of BDNet and baseline models, where parameters are iteratively updated based on the loss function. (F) Illustration of the proposed MASA loss between predicted outputs and ground truth.

The OCE dataset is acquired in three dimensions (axial × lateral × time) in Figure 2A. Prior to model training, surface flattening is performed to compensate for sample curvature. The flattened structural images (Figure 2B) are then manually annotated by two experts based on the sample configuration, as shown in Figure 2C. Following surface flattening in the spatial domain, depth‐resolved phase data are generated in Figure 2D. To facilitate stable optimization, soft labels are employed for boundary representation. Specifically, boundary locations are modelled as Gaussian distributions centered along the manually annotated interface lines, with a standard deviation of *σ* = 10. The resulting label values range continuously from 1 to 0, forming a probabilistic heatmap. Each row (1 × 600) in the heatmap corresponds to the ground truth boundary distribution at a specific depth, aligned with the corresponding lateral–temporal phase data. After preprocessing, the phase data are fed into the proposed BDNet, as well as baseline models in Figure 2E, for boundary prediction. The network outputs are compared with the ground truth heatmaps using the MASA loss function in Figure 2F. Model parameters are iteratively updated through backpropagation to minimize the loss until convergence.

### 2.4 Quantification Metric

The mean absolute error (MAE) is used to evaluate the discrepancy between the predicted and ground truth boundary positions. For each depth, the predicted boundary location is determined by identifying the peak value in the output probability distribution. To improve robustness against noise, a local regression using a weighted least‐squares smoothing function is applied to the predicted boundary positions.

(6) $$\mathrm{MAE}=\frac{1}{H}\sum_{i=1}^{H}\left|p_{i}-y_{i}\right|$$

where H denotes the number of lateral pixels in the cross‐sectional image, $p_{i}$ represents the predicted boundary position at depth index i and $y_{i}$ indicates the corresponding ground truth.

## 3 Material

To evaluate the accuracy of the proposed network, experiments were conducted on agar phantoms with well‐defined internal boundaries (Table 1). A concentration of 1.5% agar was selected as the base material, as its stiffness is close to that of normal facial skin. Variations in inclusion concentration were used to simulate different stages of tissue stiffness changes. Homogeneous agar phantoms (Fisher Scientific, No. A/1080/53) with different concentrations were fabricated to form lateral heterogeneity with either single or dual boundaries. For the dual boundaries sample, the thickness of the middle layer is around 0.5 mm. To enhance optical scattering for OCE imaging, 1% TiO_2_ was added to all samples. The stiffness of the agar phantoms was measured using a mechanical testing system (CellScale UniVert S2, CellScale, Canada) in compression mode. Detailed measurement procedures are provided in our previous work [20]. For in vivo experiments, 10 volunteers (aged 20–30 years) participated in this study to evaluate the performance of the proposed method on human acne. Ethical approval was obtained from the Physical Sciences Ethics Committee (PSEC) of the University of York, and all procedures adhered to the Declaration of Helsinki. Measurements were repeated at multiple facial locations to account for spatial variability.

**TABLE 1:** Composition of the agar phantom. Concentrations (C) are expressed as agar weight per water volume (w/v %).

Case	Amounts	Property	Left	Middle	Right
Single	6	C. (%)	1.5	—	2.5
		E (kPa)	45.90 ± 8.16	—	—
Single	6	C (%)	2.5	—	1.5
		E (kPa)	289.07 ± 20.24	—	—
Dual	5	C (%)	1.5	2.5	1.5
		E (kPa)	—	—	—
Dual	5	C (%)	1.5	2.0	1.5
		E (kPa)	—	106.76 ± 18.14	—
Dual	5	C (%)	1.5	1.7	1.5
		E (kPa)	—	78.21 ± 5.95	—
Dual	5	C (%)	1.5	1.6	1.5
		E (kPa)	—	63.61 ± 13.63	—

*Note:* The Young's modulus (E) of agar phantoms are expressed as mean ±std.

## 4 Experiment

### 4.1 The OCE System Setup

**FIGURE 3:**
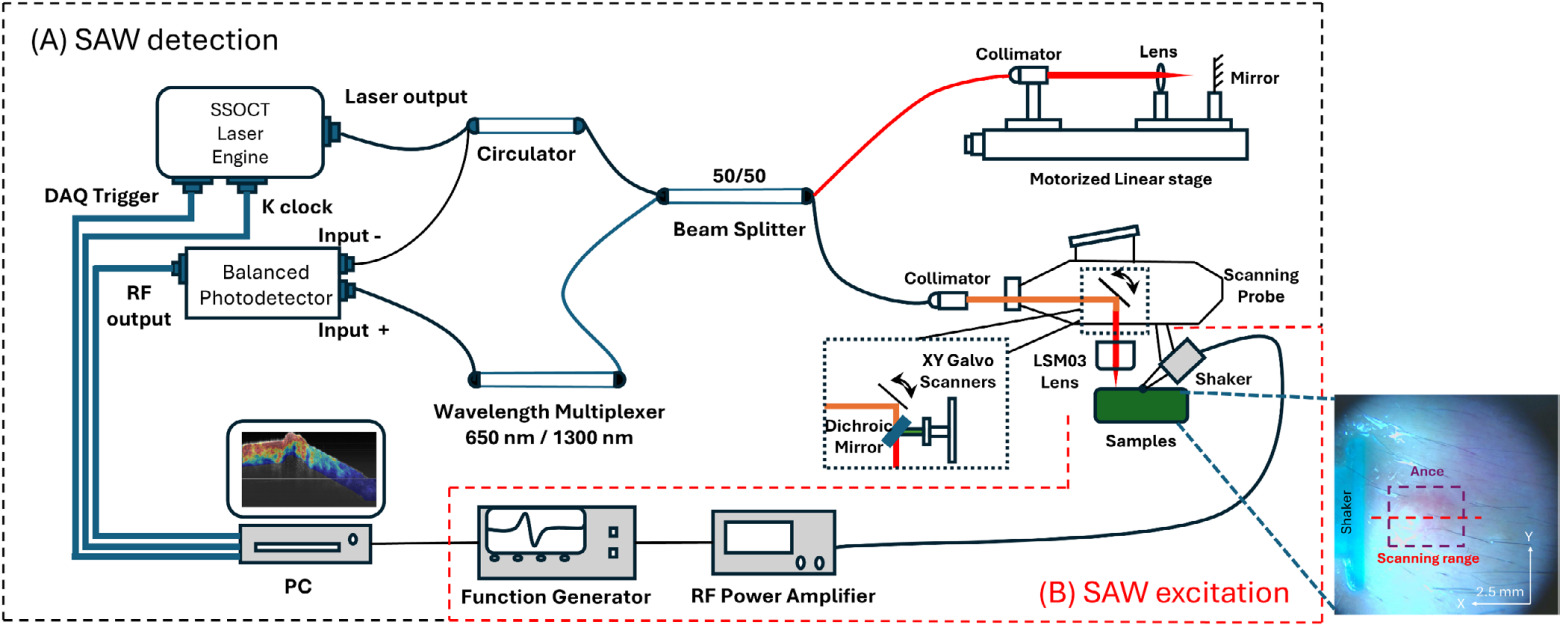
Schematic of the optical coherence elastography (OCE) experimental setup, consisting of (A) SAW detection based on swept‐source OCT and (B) SAW excitation using a contact‐based piezoelectric shaker.

The OCE system used in this study consists of two primary parts, as illustrated in Figure 3: a swept‐source optical coherence tomography (SS‐OCT) imaging module and a surface acoustic wave (SAW) excitation module [31]. The SS‐OCT system operates at a sweep rate of 400 kHz, with a central wavelength of 1300 nm and a bandwidth of 100 nm. The system achieves an axial resolution of 4.7 μm and a penetration depth of approximately 2 mm within the sample. To capture the propagation of SAWs within a two‐dimensional cross‐sectional region, an M–B scanning protocol was employed. In this configuration, the scanning beam defines a lateral field of view of 5.1 mm with a spatial resolution of 16 μm. The acquired OCE dataset forms a three‐dimensional spatiotemporal volume with dimensions of 384×600×600 (axial × lateral × time). For mechanical excitation, a square‐wave signal at 4 kHz with an amplitude of 10 Vpp was applied to drive a mechanical shaker, thereby generating surface acoustic waves propagating along the sample surface.

### 4.2 Deep Learning Implementation and Training Strategy

The deep learning pipeline was implemented in Python 3.10.19 using PyTorch 2.1.1 with CUDA 11.8, running on Linux. Model training was conducted on a workstation equipped with an NVIDIA GeForce RTX 3090 GPU. All models were optimized using the proposed MASA loss function in conjunction with the Adam optimizer, which was selected for its fast convergence and robustness to gradient scaling. The initial learning rate was set to $1\times 10^{-3}$, with a batch size of 32. Each model was trained for up to 200 epochs. To mitigate overfitting, early stopping was employed with a patience of 15 epochs based on the validation loss. The training process involved three datasets: a single‐boundary dataset, a dual‐boundary agar dataset, and a dual‐boundary acne dataset. For the single‐boundary dataset, 120 datasets (12 000 phase images) were collected. These were split into training, validation, and test sets in a ratio of 8:1:1, corresponding to 96, 12, and 12 datasets, respectively. For the dual‐boundary agar dataset, 200 datasets (20 000 phase images) were acquired and divided using the same 8:1:1 ratio, resulting in 160 training, 20 validation, and 20 test datasets. For the dual‐boundary acne dataset, 50 datasets (5000 phase images) were available, among which 40 datasets were used for training and validation, and 10 datasets (1000 images) were reserved for testing. Due to the limited size and increased complexity of the acne dataset, a transfer learning strategy was adopted. The model was first trained on the single/dual‐boundary agar dataset and subsequently fine‐tuned on the acne dataset using pretrained weights.

## 5 Results

### 5.1 Intensity Based Boundary Identification

**FIGURE 4:**
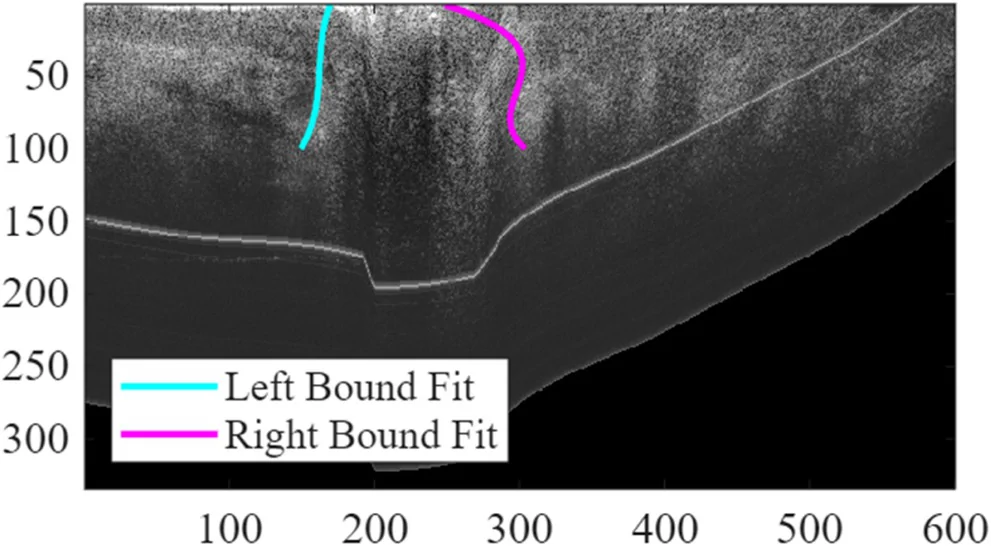
Intensity‐based boundary identification in OCT images of acne lesions.

We evaluated a traditional intensity‐based method for boundary detection using a surface‐flattened OCT structural image of a human facial acne sample. This method identifies sharp intensity shifts using a preset intensity ratio, searching inward from opposite sides at each depth. As illustrated in Figure 4, intensity variations alone are insufficient for accurate boundary placement, demonstrating the importance of integrating elastography maps to identify biomechanical stiffness contrasts.

### 5.2 Homogeneous Agar Results

**FIGURE 5:**
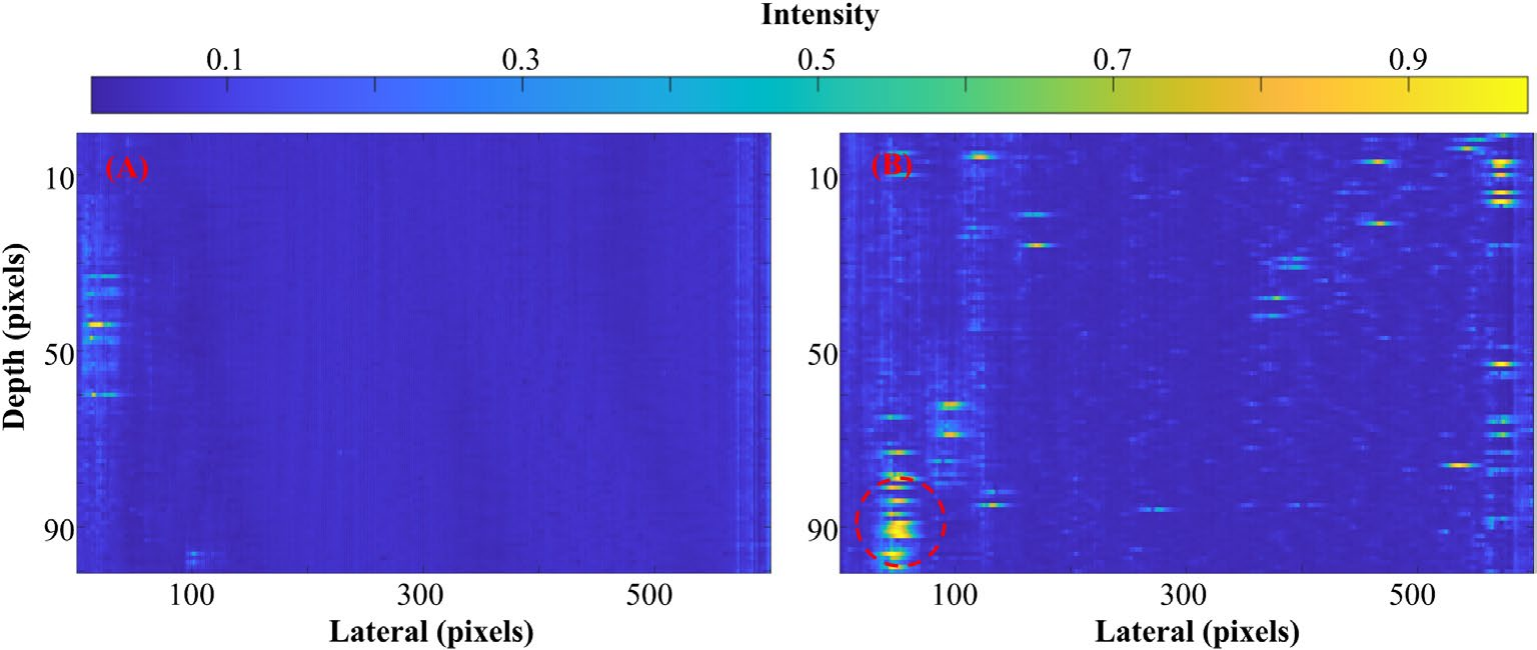
BDNet prediction maps for flattened homogeneous agar phantoms: (A) 1.5% agar phantom and (B) 1.5% agar phantom with an artificial boundary. The maps represent boundary detection responses estimated by BDNet across depth layers 1–100.

Homogeneous agar phantoms were used as a preliminary experiment to evaluate whether BDNet produces false boundary detections due to wave distortion. The prediction results for homogeneous agar, with and without an artificial boundary line, are shown in Figures 5A,B, respectively. The artificial boundary line refers to the interface formed during the layer‐by‐layer casting process of the agar phantoms. No high‐intensity responses are observed in either case, indicating that the network does not falsely interpret phase variations as boundaries. A few misidentified points can be observed at greater depths (red dashed circle in Figure 5B), which are likely caused by low signal‐to‐noise ratio (SNR). Overall, these results suggest that BDNet does not introduce significant false‐positive boundary detections in homogeneous media.

### 5.3 Single‐Boundary Agar Results

**FIGURE 6:**
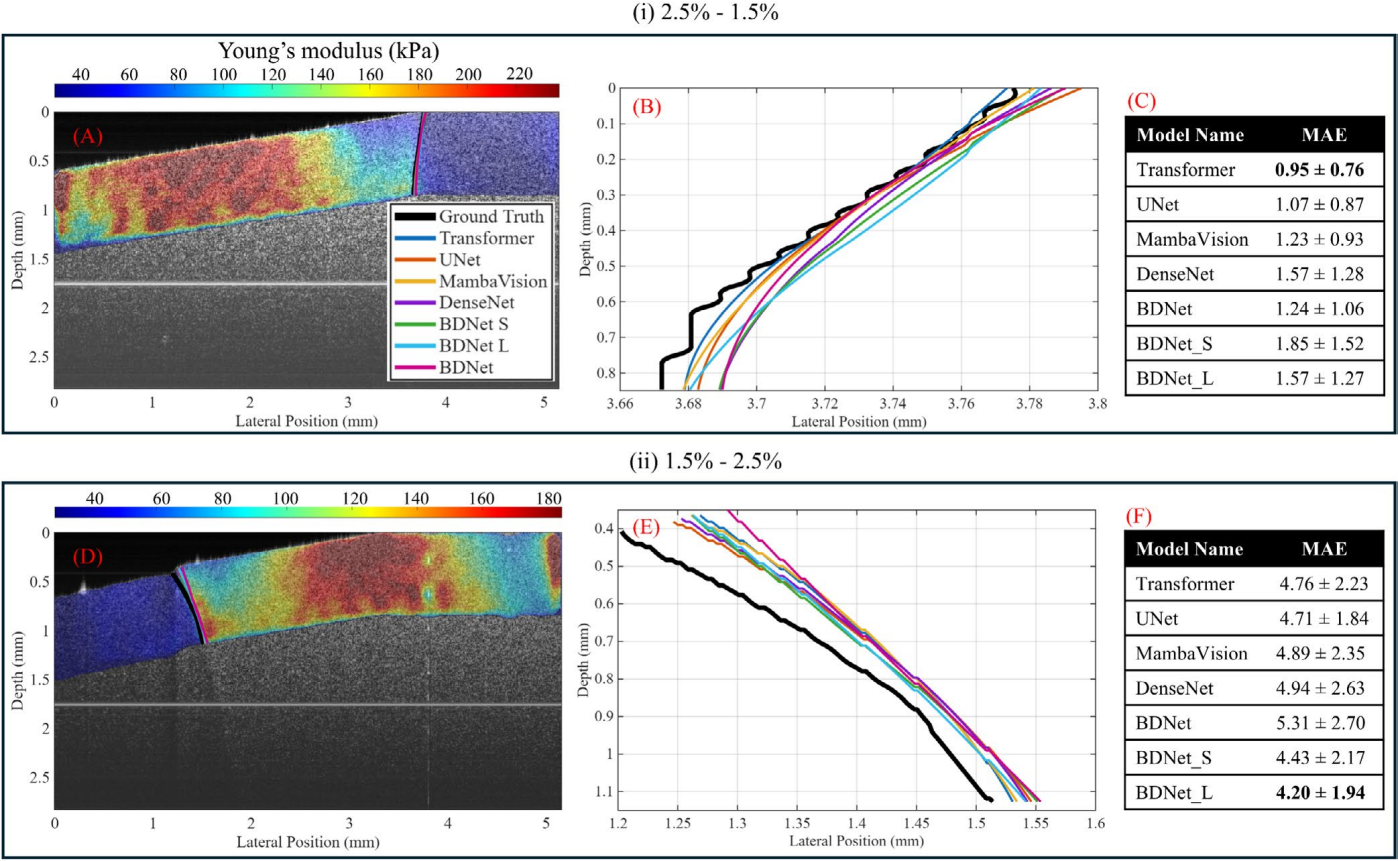
Single‐boundary agar phantom results under two concentration configurations. (i) 2.5% and 1.5% agar from left to right, and (ii) the reversed configuration. (A), (D) Cross‐sectional structural images with ground truth and predicted boundary lines overlaid for BDNet and baseline models. (B), (E) Visualization of predicted boundary locations compared with the ground truth. (C), (F) Quantitative evaluation of boundary localization using. Bold values indicate the lowest MAE.

To evaluate the feasibility of the proposed pipeline for boundary localization, BDNet and several baseline models were tested on single‐boundary agar phantoms. Performance was quantified using the MAE in pixel distance between the predicted boundary and the ground truth. Representative examples with different configurations are shown in Figure 6i and ii. In Figure 6i, the phantom consists of 2.5% and 1.5% agar from left to right, with the boundary located at approximately 3.5 mm. The corresponding Young's modulus (E) map, estimated based on previous work and overlaid on the structural image [2]. In this case, the Transformer model achieved the lowest MAE of 0.95 ± 0.76 pixels, while other models demonstrated comparable performance in Figure 6C. In Figure 6ii, the concentration distribution is reversed. A clearer stiffness contrast is observed at the boundary (Figure 6D), and the corresponding prediction is shown in Figure 6E. In this configuration, BDNet_L achieved the best performance with an MAE of 4.20 ± 1.94 pixels, corresponding to approximately 0.05 mm lateral deviation from the ground truth.

All models achieve comparable performance on the single‐boundary agar test set, with MAE values within a narrow range, as summarized in Table 2. Among them, UNet achieves the lowest MAE (2.60 ± 2.06 pixels), while DenseNet exhibits the highest error (2.96 ± 2.29 pixels). The proposed BDNet demonstrates competitive performance with an MAE of 2.75 ± 2.14 pixels. These results indicate that, for simple structures with clear lateral contrast, all models are capable of accurately identifying boundaries associated with sharp changes in wave propagation. Furthermore, the consistency between the predicted boundaries, structural images, and elastogram maps suggests that the proposed pipeline provides reliable boundary localization.

**TABLE 2:** Boundary localization performance on the single‐boundary agar test set (MAE in pixels).

Model name	MAE (mean ± std., pixels)
DenseNet	2.96 ± 2.29
MambaVision	2.83 ± 2.22
UNet	**2.60 ± 2.06**
Transformer	2.92 ± 2.20
BDNet	2.75 ± 2.14
BDNet_S	2.81 ± 2.20
BDNet_L	2.73 ± 1.97

*Note:* Bold fonts indicate the lowest MAE.

### 5.4 Dual‐Boundary Agar Results

**FIGURE 7:**
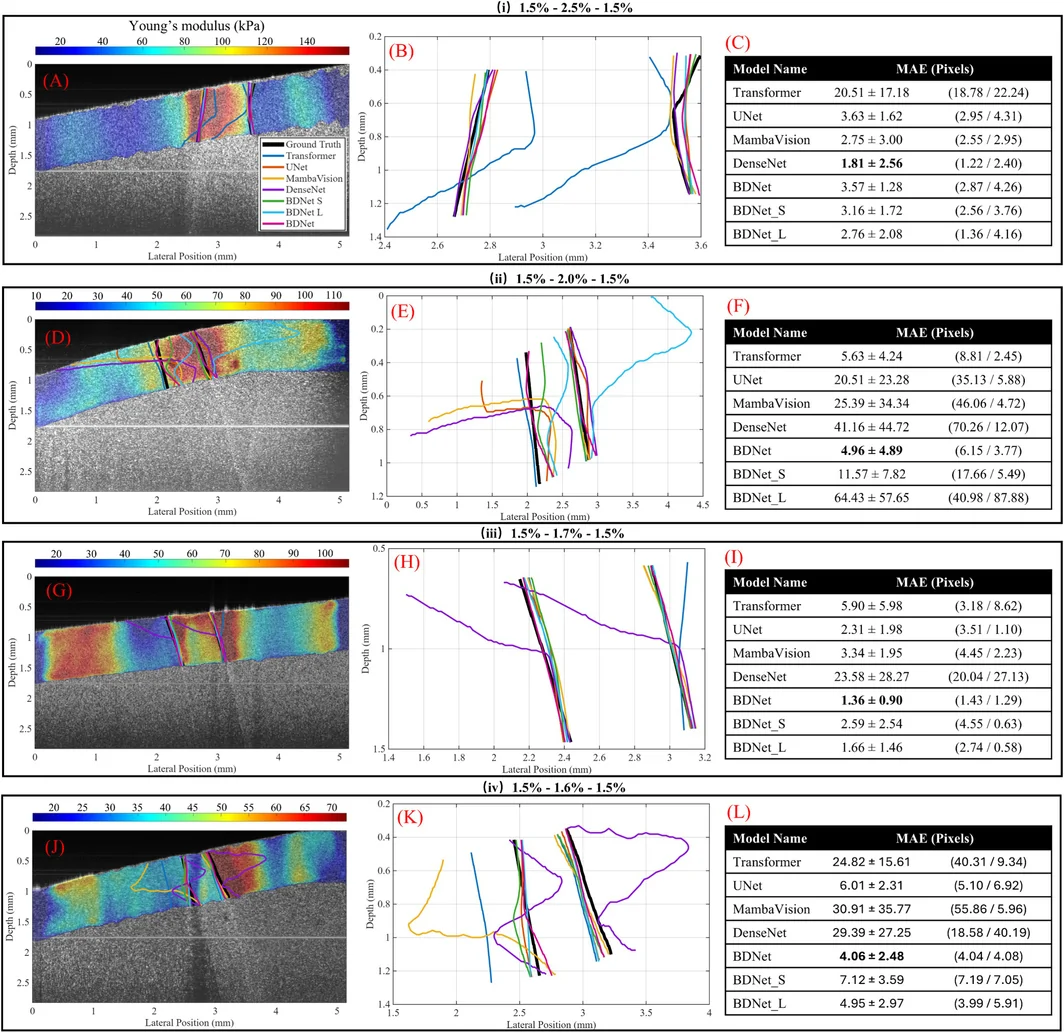
Results for dual‐boundary agar phantoms with varying middle‐layer concentrations: (i) 2.5%, (ii) 2.0%, (iii) 1.7%, and (iv) 1.6%. (A), (D), (G), (J) Cross‐sectional structural images overlaid with stiffness maps, ground truth, and predicted boundary lines from BDNet and baseline models. (B), (E), (H), (K) Visualization of predicted boundary locations compared with ground truth. (C), (F), (I), (L) Quantitative evaluation using MAE. The first column shows the average MAE, while the second column reports MAE for left and right boundaries separately. Bold values indicate the best performance.

The results for dual‐boundary agar phantoms with varying middle‐layer concentrations are shown in Figure 7i–iv, where the inclusion concentration decreases from 2.5% to 1.6%. In the high contrast case (≈240 kPa difference) in Figure 7i, the stiffness map shows a leftward spatial shift relative to the structural boundary (Figure 7A), likely caused by strong wave distortion due to the large stiffness contrast. In this scenario, the Transformer shows inferior performance, with underestimated inclusion width (Figure 7C), whereas other models achieve comparable accuracy, with DenseNet yielding the best results. For the moderate contrast case (≈60 kPa difference) in Figure 7ii, the stiffness map is shifted in the rightward direction, indicating instability in elastography‐based localization. Several models, including DenseNet, MambaVision, UNet, and BDNet_L, fail to accurately detect boundaries near the surface (within approximately 0.2 mm depth). In contrast, BDNet and Transformer maintain relatively stable performance with MAE values below 6 pixels across depth.

In the lower contrast case (≈30 kPa difference) in Figure 6iii, the stiffness map fails to preserve the integrity of the inclusion, despite reasonable elasticity values. DenseNet again shows inaccurate boundary estimation near the surface (Figure 6H), although performance improves at greater depths. Other models demonstrate high accuracy under this condition. In the extremely low‐contrast case (≈15 kPa difference) in Figure 6iv, where the inclusion and background concentrations are very similar, the stiffness map becomes severely misaligned with the structural image. Only UNet, BDNet, and its variants are able to partially recover the boundary locations, while MambaVision exhibits degraded performance, particularly due to left boundary offsets. Overall, these results demonstrate that boundary localization becomes increasingly challenging as stiffness contrast decreases. The proposed BDNet shows more robust performance across varying contrast conditions, particularly in cases where elastography‐based methods suffer from distortion or misalignment.

Across the entire test dataset with different agar concentrations paired with the 1.5% background (Table 3), DenseNet demonstrates the lowest performance, accompanied by a relatively large standard deviation, indicating poor robustness. MambaVision and Transformer‐based models show degraded performance under low stiffness contrast conditions (e.g., 1.6% and 1.7%), suggesting limited sensitivity to subtle boundary variations. In contrast, UNet, BDNet, and its variants achieve more stable and accurate results across most cases. Notably, BDNet maintains high localization accuracy for low contrast inclusions (1.6% and 1.7%), demonstrating its robustness in challenging scenarios. A noticeable performance gap between UNet and BDNet is observed in the 2.0% case, where BDNet achieves superior boundary localization accuracy. These results highlight the effectiveness of BDNet in capturing subtle stiffness variations for accurate boundary detection.

**TABLE 3:** Boundary localization performance (MAE) on the dual‐boundary agar dataset.

Model name	MAE (mean ± std., pixels)			
	1.6%	1.7%	2.0%	2.5%
DenseNet	11.64 ± 20.21	11.36 ± 18.63	19.28 ± 31.43	10.49 ± 19.59
MambaVision	17.24 ± 27.87	3.82 ± 3.05	14.79 ± 22.39	9.41 ± 12.38
UNet	3.63 ± 2.50	3.88 ± 2.43	11.27 ± 17.38	**5.05 ± 5.98**
Transformer	39.83 ± 29.80	9.71 ± 7.28	24.61 ± 24.09	11.04 ± 12.01
BDNet	**2.72 ± 2.23**	3.46 ± 4.19	**5.74 ± 4.08**	5.38 ± 7.93
BDNet_S	3.97 ± 3.37	4.44 ± 3.40	8.35 ± 7.22	15.64 ± 44.20
BDNet_L	2.64 ± 2.50	**3.32 ± 2.86**	26.16 ± 43.56	5.54 ± 6.54

*Note:* Bold fonts indicate the lowest MAE. Concentration denotes inclusion values paired with a 1.5% agar background.

### 5.5 Dual‐Boundary Acne Results

**FIGURE 8:**
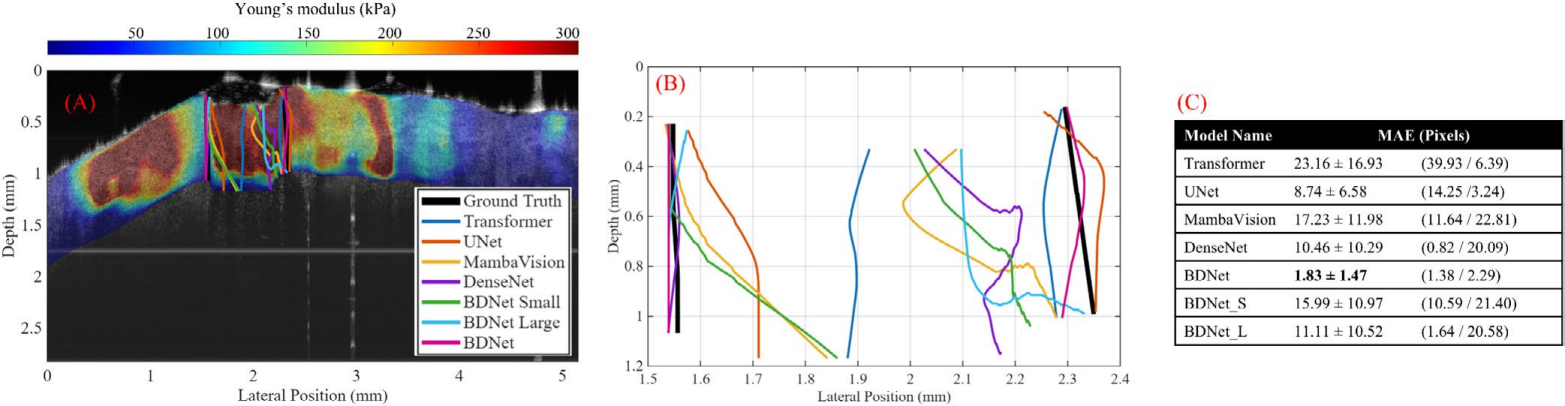
Dual‐boundary human skin acne results. (A) Cross‐sectional structural image overlaid with ground truth and predicted boundaries from BDNet and baseline models. (B) Zoomed‐in view of predicted boundary locations compared with ground truth. (C) Quantitative comparison of MAE between ground truth and model predictions.

Acne presents a more challenging scenario due to increased attenuation of both wave propagation and optical signals, resulting in phase images with lower SNR. Figure 8A shows the structural image overlaid with stiffness information and the predicted boundaries from different models. The acne region is located at approximately 2 mm along the lateral direction and exhibits an indented morphology compared to the surrounding normal tissue. As illustrated in Figure 8B,C, DenseNet and BDNet achieve relatively accurate localization of the left boundary; however, DenseNet exhibits a significant offset on the right boundary. In contrast, BDNet provides more consistent boundary predictions on both sides. Transformer, UNet, and MambaVision show degraded performance, particularly on the left boundary.

The quantitative results on the acne test dataset are summarized in Table 4. The models achieve an average MAE of approximately 20 pixels, indicating increased difficulty in boundary localization for in vivo data. The relatively large standard deviations suggest variability across different acne cases. Among the evaluated methods, BDNet_L achieves the highest accuracy, while the Transformer model exhibits the largest error. Despite this, the overall predictions remain clinically acceptable, as a deviation of 20 pixels is relatively small compared to the lateral extent of acne regions (approximately 600 pixels).

**TABLE 4:** Boundary localization performance on the dual‐boundary acne test dataset using MAE.

Model name	MAE (mean ± std., pixels)
DenseNet	22.39 ± 19.56
MambaVision	24.66 ± 18.66
UNet	21.08 ± 19.76
Transformer	26.49 ± 18.53
BDNet	19.11 ± 17.78
BDNet_S	21.52 ± 17.43
BDNet_L	**14.79 ± 11.14**

*Note:* Bold fonts indicate the lowest MAE.

### 5.6 Baseline Comparison

The performance of baseline and proposed models, evaluated with a batch size of 32 and an input resolution of 600 × 600 pixels, is summarized in Table 5. Most models contain fewer than 0.6 million parameters, except for BDNet_L, which exceeds 1 million parameters and correspondingly exhibits lower inference speed (FPS). In terms of computational complexity, MambaVision demonstrates relatively low FLOPs (3.08 G) due to its use of SSM. The proposed BDNet leverages this efficiency while integrating a UNet backbone, maintaining a comparable model size (0.486 M parameters) with moderate computational cost (25.60 G FLOPs) and stable inference speed (82.2 FPS). For a fair comparison, models with similar parameter scales are considered. Transformer‐based models achieve the highest inference speed (2926.4 FPS), followed by MambaVision (199.0 FPS), while CNN‐based models generally exhibit lower computational efficiency. The ablation results of BDNet indicate that the base model achieves a balanced trade‐off between accuracy and efficiency.

**TABLE 5:** Comparison of model complexity and inference efficiency for boundary localization from phase images.

Model name	Parameters (M)	FLOPs (G)	FPS
Transformer	0.551	1.04	2926.4
DenseNet	0.300	6.88	374.6
MambaVision	0.320	3.08	199.0
UNet	0.484	25.60	81.5
BDNet	0.486	25.60	82.2
BDNet_S	0.122	6.51	142.9
BDNet_L	1.092	57.28	59.4

### 5.7 Loss Function Comparation

**FIGURE 9:**
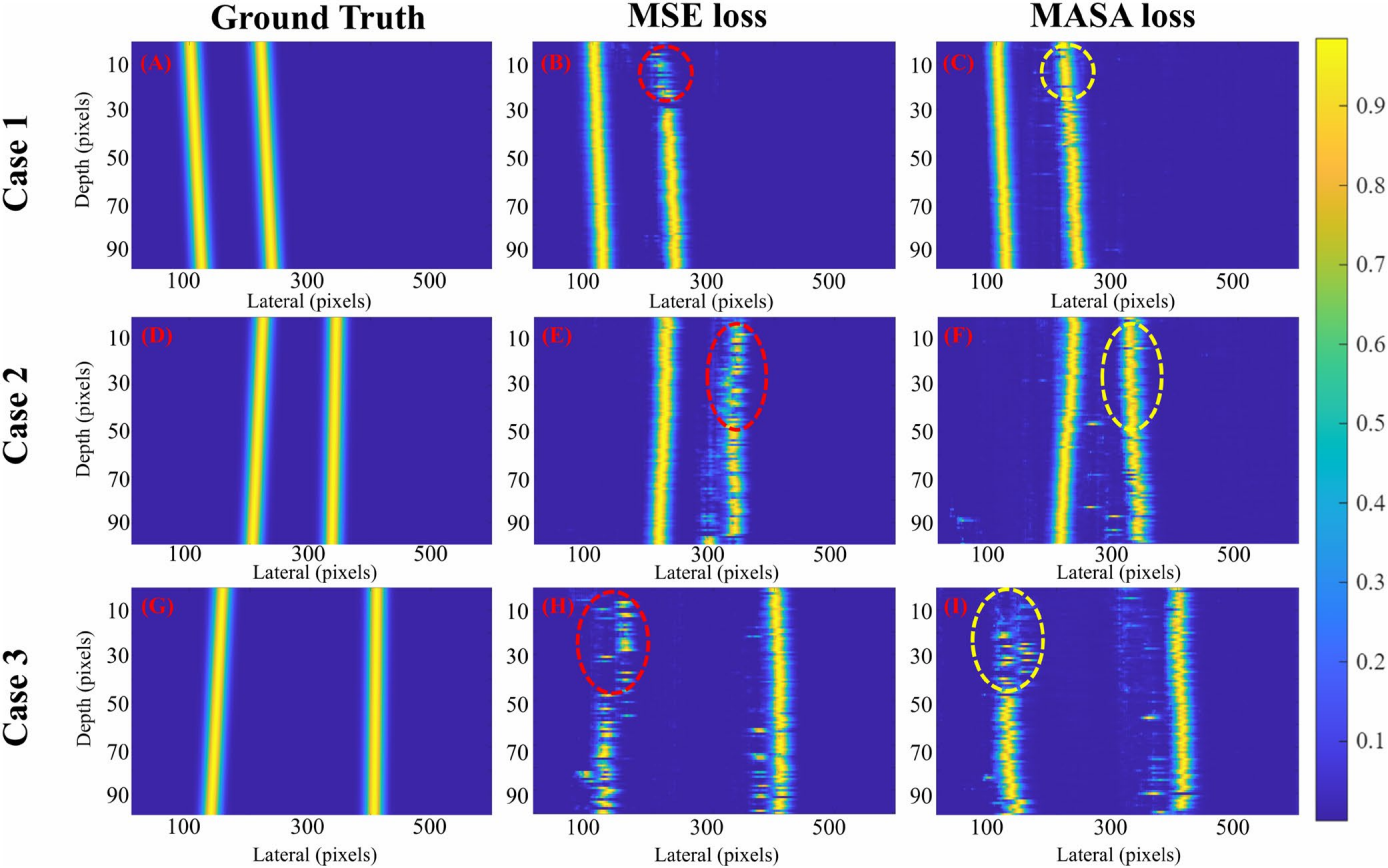
Qualitative comparison of boundary detection results on dual‐boundary agar samples. (A), (D), and (G) show the manually annotated ground truth. (B), (E), and (H) present predictions using MSE loss. (C), (F), and (I) present predictions using MASA loss.

Qualitative results further highlight the differences between the two loss functions. In several cases, predictions obtained using mean squared error (MSE) show reduced intensity at one of the boundary lines (red dashed circles in Figure 9B,E,H), indicating partial loss of boundary information. In contrast, the MASA‐based predictions (Figure 9C,F) better preserve the structural integrity of both boundaries within the corresponding regions (yellow dashed circles). Although slight discontinuities are observed in the left boundary in Figure 9I, the overall boundary trend remains identifiable due to the preserved low‐intensity responses.

**FIGURE 10:**
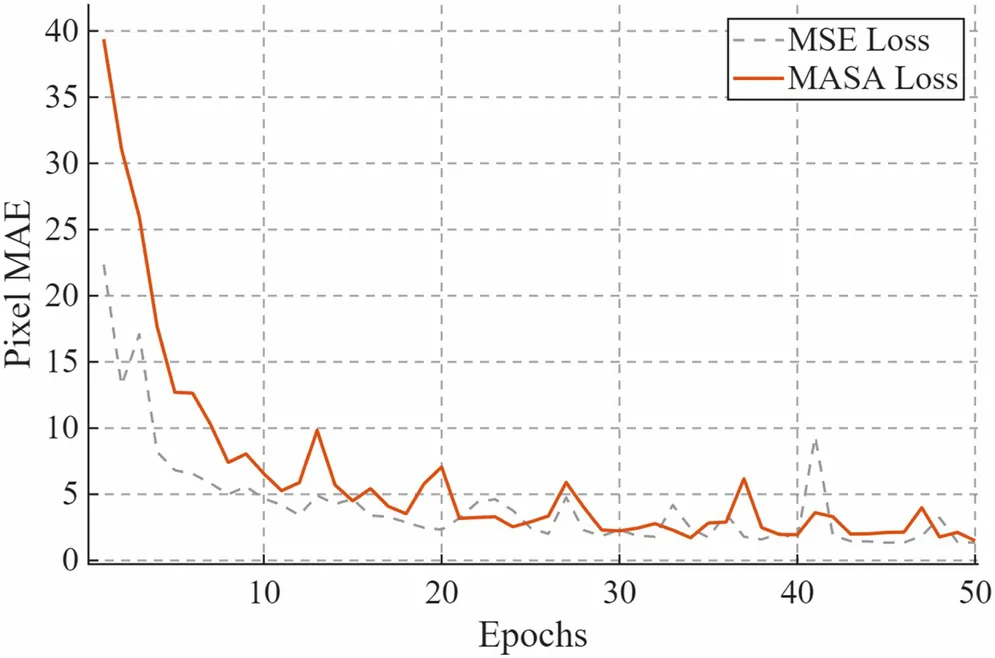
Quantitative comparison of boundary detection performance under different loss functions (MSE and MASA), measured by MAE.

A dual‐boundary agar dataset composed of 1.5% and 2.5% concentrations is used to evaluate the performance difference between the conventional MSE loss and the proposed MASA loss within BDNet. MASA exhibits slower convergence, requiring approximately 30 epochs to stabilize, whereas MSE converges in around 20 epochs, as shown in Figure 10. Despite this difference in convergence speed, both methods achieve a similar final accuracy of approximately 1.5 pixels MAE.

## 6 Discussion

The experimental results demonstrate that the proposed BDNet enables robust boundary detection in OCE across both phantom and in vivo skin data under varying stiffness contrasts and signal conditions. Compared with conventional elastography approaches, BDNet directly exploits waveform variations in phase images, bypassing the intermediate velocity or elasticity estimation steps. This avoids error accumulation associated with parameter‐dependent processing and leads to improved boundary localization accuracy and computational efficiency.

In this study, ground truth boundaries are manually annotated on 2D cross‐sectional structural images, providing a more direct and reliable reference than indirectly inferred velocity maps. To facilitate effective model training, soft labels modelled as Gaussian distributions are employed to represent boundary locations. This design enables smooth and differentiable supervision, accommodates multiple candidate boundaries, and improves optimization stability. Furthermore, the proposed MASA loss enhances boundary detection by explicitly distinguishing between foreground and background regions. This design prevents excessive suppression of physically meaningful wave responses and allows the model to focus on accurate boundary localization. The quantitative comparison between mean squared error (MSE) and MASA is shown in Figure 9. The design of the MASA loss assigns asymmetric weights to false positive and false negative predictions, aiming to preserve fine boundary details while reducing missed detections, particularly for complex boundary shapes. However, this weighting strategy introduces a trade‐off. Compared with the MSE loss function, MASA may lead to slower convergence during backpropagation due to its emphasis on multiple boundary candidates. The training process in terms of loss convergence is shown in Figure 10. In addition, by emphasizing detailed boundary responses, the model may retain a certain level of background noise in the predicted maps. For future applications, the MASA loss should be further optimized to balance boundary sensitivity, noise suppression, and computational efficiency across different imaging conditions.

From a modelling perspective, the proposed BDNet integrates a UNet backbone with a Mamba‐based state space module for wave‐based boundary detection. The UNet effectively captures local waveform variations along the lateral dimension through its hierarchical structure and skip connections, preserving high‐resolution spatial features. However, it is inherently limited in modelling long‐range dependencies [32]. To address this limitation, the Mamba module introduces an efficient sequence modelling mechanism that captures global contextual information along the lateral axis [33]. This design is consistent with the physical characteristics of wave propagation, where boundary‐related features are distributed sequentially along the lateral direction. In contrast, CNN architectures such as DenseNet and UNet primarily rely on local receptive fields, which restrict their ability to model global dependencies [34]. Transformer‐based methods address this limitation through self‐attention mechanisms, enabling global context modelling but at the cost of increased computational complexity, particularly for high‐resolution inputs. Meanwhile, MambaVision improves efficiency by leveraging SSM, but its representation capacity for this task remains limited due to the lack of explicit integration with multi‐scale spatial features [35, 36] The BDNet achieves a balance between capturing local and global features while maintaining computational efficiency.

There are several limitations in this study. First, wave distortion may occur at boundary locations due to factors beyond stiffness contrast, such as local tissue heterogeneities. These effects may introduce ambiguity, potentially leading the model to associate distortion patterns with boundaries rather than true stiffness transitions. Although homogeneous agar phantoms with artificial boundaries were used to mitigate shortcut learning, this issue cannot be completely eliminated in Figure 5. Second, the acne dataset used in this study is relatively limited in size, which may constrain the generalization capability of the model. In addition, ground‐truth boundaries were manually defined from structural images, and only clearly identifiable regions were included, limiting the amount of training data. Expanding the dataset with more diverse boundary patterns may further improve model robustness. Moreover, unlike agar phantoms, human skin is anisotropic and structurally heterogeneous. Factors such as skin texture and body hair may affect wave propagation and introduce additional variability in phase signals, highlighting the need for more diverse in vivo data. Besides, while the clinical utility of the proposed method requires further validation, the results demonstrate early translational potential for applications involving acne characterization, fibrosis or scar assessment, and tumor boundary delineation. Finally, from a computational perspective, BDNet integrates a UNet backbone with a Mamba‐based module for global context modelling, resulting in higher computational cost compared to lightweight CNN models such as DenseNet. Although its efficiency remains comparable to that of UNet and significantly lower than Transformer‐based methods in terms of computational complexity, this increased demand may still pose challenges in hardware‐limited environments.

Acne was included as a representative inflammatory skin condition with localized biomechanical changes. The ability of BDNet to identify lesion boundaries from OCE wave propagation data suggests potential utility for assessing tissue heterogeneity and lesion extent. Although these findings indicate early translational potential, further longitudinal and outcome‐based studies are required to determine clinical relevance for scar prediction or treatment stratification.

## 7 Conclusion

In this study, we proposed BDNet, a deep learning framework for direct boundary localization from OCE depth‐resolved phase images. By directly analyzing waveform variations, the proposed method bypasses conventional velocity estimation and provides a more reliable approach for identifying tissue boundaries. In summary, BDNet offers a robust and computationally efficient solution for boundary detection in elastography, with strong potential for clinical applications in dermatology, ophthalmology, and other areas requiring precise tissue characterization.

## Funding

The authors have nothing to report.

## Disclosure

The authors have nothing to report.

## Conflicts of Interest

The authors declare no conflicts of interest.

## Supporting information


**Table S1:** Configuration of BDNet variants with different base channel sizes and mamba layers.
**Table S2:** Configuration of the Transformer network (C = 16).
**Table S3:** Configuration of the UNet network (C = 16).
**Table S4:** Configuration of the Densenet network (C = 32).
**Table S5:** Configuration of the MambaVision network (C = 64).

## Data Availability

Data underlying the results presented in this paper are not publicly available at this time but may be obtained from the authors upon reasonable request.
