# Supplementary material for: Deep Learning‐Based Inclusion Boundary Identification Using Wave Propagation in Optical Coherence Elastography
Source: J Biophotonics. 2026 Aug 23;19(8):e70345. doi: 10.1002/jbio.70345 (PMC13500869; doi:10.1002/jbio.70345)
Supplement: Supplementary file 1 [file JBIO-19-e70345-s001.docx]

**Supplement for “Deep Learning-Based Inclusion Boundary Identification Using Wave Propagation In Optical Coherence Elastography”**

This supplement material presents the construction of BDNet variants.

A. **Ablation Study of BDNet**

To balance model accuracy and computational efficiency, several variants of the proposed BDNet were designed with different base channel sizes and numbers of Mamba layers. The network adopts a UNet backbone integrated with a Mamba-based sequence modelling module for regression on single-channel phase images. Given the input resolution of 600 × 600, increasing the channel width significantly raises computational cost and memory usage. Therefore, the base channel size was limited to a maximum of 24.

Based on this design, three variants: BDNet_S, BDNet, and BDNet_L, were constructed, as summarized in Table S1. These models differ in both base channel size and Mamba depth, enabling systematic evaluation of the trade-off between model complexity and performance. The structural configurations of the Transformer, UNet, DenseNet, and MambaVision models are summarized respectively in Tables S2-S5.

Table S1. Configuration of BDNet variants with different base channel sizes and mamba layers

| Model Variant | Base Channels (C) | Mamba Layers |
| --- | --- | --- |
| BDNet_S | 8 | 1 |
| BDNet | 16 | 2 |
| BDNet_L | 24 | 3 |

Table S2. Configuration of the Transformer network (C =16)

| Stage / Layer | Specifications | Output |
| --- | --- | --- |
| Input | Phase Image | 1, 600, 600 |
| Patch Embed | 2 × [Conv2D + BN + ReLU], Stride=2 | 16, 150, 150 |
| Asymmetric Extractor | 3 × [Conv2D + BN + ReLU], Stride=(1, 2) | 128, 150, 19 |
| Time Pooling | Adaptive MaxPool2D | 150, 128 |
| Encoder | 2 ×Transformer Layers, Heads=4, PE | 150, 128 |
| Decoder | MLP + 1D Interpolation | 600 |

Table S3. Configuration of the UNet network (C = 16)

| Stage / Layer | Specifications | Output |
| --- | --- | --- |
| Input | Phase Image | 1, 600, 600 |
| Encoder | 2 × [MaxPool2D + DoubleConv] | 128, 75, 75 |
| Decoder | 3 × [ConvTranspose2D + DoubleConv] | 16, 600, 600 |
| Time Pooling | Adaptive MaxPool2D | 16, 600 |
| Regression | 2 × Conv1D + GELU + Sigmoid | 600 |

Table S4. Configuration of the Densenet network (C = 32)

| Stage / Layer | Specifications | Output |
| --- | --- | --- |
| Input | Phase Image | 1, 600, 600 |
| Stem | 2 × [Conv2D + BN + ReLU], Stride=2 | 32, 150, 150 |
| Dense BackBone | 3 × DenseBlock | 88, 150, 19 |
| Time Pooling | Adaptive MaxPool2D | 150, 88 |
| MLP | Linear + GELU + Linear | 150 |
| Rescale | 1D Interpolation | 600 |

Table S5. Configuration of the MambaVision network (C = 64)

| Stage / Layer | Specifications | Output |
| --- | --- | --- |
| Input | Phase Image | 1, 600, 600 |
| Patch Embed | 2 × Conv2D | 64, 150, 150 |
| MambaVision Blocks | 4 Stages (depths= [1,1,2,1]) | 512, 150, 19 |
| Time Pooling | Adaptive MaxPool2D | 150, 512 |
| MLP | Linear + GELU + Linear | 150 |
| Rescale | 1D Interpolation | 600 |
